# Nickel Availability in Soil as Influenced by Liming and Its Role in Soybean Nitrogen Metabolism

**DOI:** 10.3389/fpls.2016.01358

**Published:** 2016-09-08

**Authors:** Fernando G. de Macedo, Joana D. Bresolin, Elcio F. Santos, Felipe Furlan, Wilson T. Lopes da Silva, Joe C. Polacco, José Lavres

**Affiliations:** ^1^Center for Nuclear Energy in Agriculture, University of Sao PauloPiracicaba, Brazil; ^2^Brazilian Agricultural Research CorporationSão Carlos, Brazil; ^3^University of Missouri, ColumbiaMO, USA

**Keywords:** base-cation saturation, grain nitrogen accumulation, micronutrient, nickel, nitrate reductase, soil rhizosphere, urease

## Abstract

Nickel (Ni) availability in soil varies as a function of pH. Plants require Ni in small quantities for normal development, especially in legumes due its role in nitrogen (N) metabolism. This study investigated the effect of soil base saturation, and Ni amendments on Ni uptake, N accumulation in the leaves and grains, as well as to evaluate organic acids changes in soybean. In addition, two N assimilation enzymes were assayed: nitrate reductase (NR) and Ni-dependent urease. Soybean plants inoculated with *Bradyrhizobium japonicum* were cultivated in soil-filled pots under two base-cation saturation (BCS) ratios (50 and 70%) and five Ni rates – 0.0; 0.1; 0.5; 1.0; and 10.0 mg dm^-3^ Ni. At flowering (R1 developmental stage), plants for each condition were evaluated for organic acids (oxalic, malonic, succinic, malic, tartaric, fumaric, oxaloacetic, citric and lactic) levels as well as the activities of urease and NR. At the end of the growth period (R7 developmental stage – grain maturity), grain N and Ni accumulations were determined. The available soil-Ni in rhizosphere extracted by DTPA increased with Ni rates, notably in BCS50. The highest concentrations of organic acid and N occurred in BCS70 and 0.5 mg dm^-3^ of Ni. There were no significant differences for urease activity taken on plants grown at BSC50 for Ni rates, except for the control treatment, while plants cultivated at soil BCS70 increased the urease activity up to 0.5 mg dm^-3^ of Ni. In addition, the highest values for urease activities were reached from the 0.5 mg dm^-3^ of Ni rate for both BCS treatments. The NR activity was not affected by any treatment indicating good biological nitrogen fixation (BNF) for all plants. The reddish color of the nodules increased with Ni rates in both BCS50 and 70, also confirms the good BNF due to Ni availability. The optimal development of soybean occurs in BCS70, but requires an extra Ni supply for the production of organic acids and for increased N-shoot and grain accumulation.

## Introduction

Nickel (Ni), is the 22nd most abundant element in the earth’s crust and is found in natural soils in trace concentrations (Hussain et al., 2013). It is an essential element for plants and for many bacteria (Brown, 2007). In bacteria Ni is present in several enzymes, including urease, glyoxalase-I, hydrogenases, some superoxide dismutases, carbon monoxide dehydrogenase and methyl-coenzyme M reductase (Ragsdale, 1998). Until recently urease was the only known Ni-containing enzyme in higher plants (Polacco et al., 2013), but Mustafiz et al. (2014) reported that plant glyoxalase-I requires Ni for maximal activity. While glyoxalase-I appears to be activated by Ni *in vitro* (Mustafiz et al., 2014), in the absence of other proteins, Ni activation of urease requires three accessory proteins. The assembly of the urease Ni metallocenter is not yet clearly understood (Carter et al., 2009; Witte, 2011).

The first Ni deficiency reports in plants date from 1918, with the symptom known as “mouse ear,” that describes the severe developmental disorder presented by young leaves of pecan trees (*Carya illinoensis*; Wood et al., 2004a). The authors identified possible alterations in ureide, amino acid and organic acid metabolism (Wood et al., 2004b; Bai et al., 2006). Brown (2007) suggested accumulation of nitrate as a consequence of the low malate dehydrogenase activity under Ni deprivation. This enzyme is purported to provide energy for the nitrate reduction process. Alibakhshi and Khoshgoftarmanesh (2015) reported that small amounts Ni can reduce the nitrate concentration of plants by increasing the activity of nitrate reductase (NR). Leaf tip necrosis was also identified by Eskew et al. (1983) in Ni-deficient soybean. This deficiency reduces the action of urease, causing the accumulation of urea and organic acids (oxalic and lactic; Bai et al., 2006).

In the case of Ni toxicity, several studies reported Ni toxicity symptoms in plants such as: low nutrient uptake (Charles and Issac, 2014; Fabiano et al., 2015); nutrient imbalances (Saad et al., 2016); reduction in seed yield (Piccini and Malavolta, 1992); decreased chlorophyll in leaves of coffee (Pavan and Bingham, 1982) and soybean (Sirhindi et al., 2016); and decreased stomatal conductance (Velikova et al., 2011). Phytotoxicity by Ni is a result of its action in the photosystems, disturbing the Calvin cycle and inhibiting electron transport chains because of excessive amounts of ATP and NADPH accumulated as a result of inefficient biochemical pathway reactions (Krupa et al., 1993; Yusuf et al., 2011).

Some plant micronutrients, such as Ni, are often challenging, partly because they have a narrow dynamic range between minimal requirement and toxicity, and partly because their concentrations and chemical speciation are subject to major fluctuations in the soil (Krämer et al., 2007). Although Ni is a recognized essential mineral nutrient element for higher plants, its agricultural and biological significance is poorly understood (Bai et al., 2006).

In biological nitrogen fixation (BNF) Ni is an essential component of the hydrogenase (Ni-Fe) that recycles H_2_, an obligatory product of N_2_ reduction (González-Guerrero et al., 2014). *Frankia* root nodules, more than leaves, concentrate Ni (Wheeler et al., 2001). The availability of Ni in soil is mainly variable by soil characteristics (Soares et al., 2011) and the effect of liming (Berton et al., 2006). Ni availability in soil is also reduced by increasing the base-cation saturation (BCS) which consequently raises the pH (Sreekanth et al., 2013), this is common for all cationic micronutrients. Some studies with soybean in tropical soils have shown that the elevation of BCS through liming has led to micronutrient deficiencies (Novais et al., 1989; Caires et al., 2003), notably those of Zn and Mn. However, these studies did not investigate Ni availability.

Since Eskew et al. (1983) noted the importance of Ni to soybean, whatever the N source (nitrate, ammonia or BNF), several studies have been conducted in order to evaluate the role Ni in the N cycle (Zornoza et al., 1999; Bybordi and Gheibi, 2009; Gheibi et al., 2009; Khoshgoftarmanesh et al., 2011; Kutman et al., 2013, 2014; Alibakhshi and Khoshgoftarmanesh, 2015). Notably, urease and NR activities have been evaluated in order to establish the role of Ni in plant N-metabolism. Despite of Ni supply enhances NR activity in cucumber (Tabatabaei, 2009), tomato (Gad et al., 2007) and onion (Alibakhshi and Khoshgoftarmanesh, 2015), Ni-excess inhibits this enzyme (Mishra and Dubey, 2011). In addition, it has been pointed out that urease activity increases in response to soybean seed treatment with Ni (Lavres et al., 2016) or to increased Ni content in the grains (Kutman et al., 2013, 2014), resulting in the N- remobilization from the old to new leaves.

The decrease of Ni availability in soil by liming might influence N-plant nutrition, metabolism of organic acids and enzymes related to nitrogen. To test this hypothesis soil pH was varied indirectly by using two base-cation saturation ratios (BCS ratios). The objective was to identify influences of Ni on urease and NR activities, N, Ni, and organic acids (oxalic, malonic, succinic, malic, tartaric, fumaric, oxaloacetic, citric and lactic) concentration in tissues of soybean.

## Materials and Methods

### Experiment Description

Soybean plants were grown under greenhouse conditions. Plants were grown in pots with capacity of 10 dm^-3^. Temperature maximum and minimum were 35 and 18°C, respectively. Pots were filled with soil from the 0 – 20 cm layer of Alfisol.

Seventy pots were distributed with seven replicates per treatment, using factorial a 2 × 5 design (50 and 70% BCS and five doses of Ni: 0, 0.1, 0.5, 1.0, and 10.0 mg dm^-3^ of soil). Ni rates were established according to the results of Dalton et al. (1985); Atta-Aly (1999), Kevresan et al. (2001) and Milošević et al. (2002).

### Experimental Design

#### Plant Cultivation

The pots were filled with 8 dm^-3^ soil (whose density is 1.1 kg dm^-3^) collected from the 0–20 cm layer of Alfisol with 51 % clay, 18 % silt and 31 % sand. Its physico-chemical characteristics were: pH = 5; organic matter = 40 g dm^-3^; P = 20 mg dm^-3^; K = 2.9 mmol dm^-3^; Ca = 33 mmol dm^-3^; Mg = 10 mmol dm^-3^; H + Al = 53 mmol dm^-3^; Al = 1 mmol dm^-3^ and Ni_(DTPA)_ = 0.04 mg dm^-3^. Soil pH was determined with 0.01 mol L^-1^ CaCl_2_ (ratio of 1:2.5 for soil and solution – w/v), according Raij et al. (2001). Available P, K, Ca and Mg were extracted by ion-exchange resins, and then quantified by colorimetric (P), flame photometric (K), and atomic absorption spectroscopic (Ca and Mg) methods (Raij et al., 2001). Exchangeable Al was extracted with KCl solution and then determined by titration (Raij et al., 2001). Concurrent with filling of pots, liming was performed individually, following the method of base saturation applying a calcium and magnesium carbonate mixture (molar ratio 3:1) with the dosage required to meet the respective saturation values for bases. After liming, pots were irrigated to maintain 50% of field capacity, and they remained in this condition until sowing, 30 days later. Liming provided pH 5.0 ± 0.2 to BCS50 and 5.5 ± 0.3 to BCS70.

Soybean variety *CODETEC 202*, which presents mid cycle of development and determined growth habit, was grew. The seeds were treated with a sugared solution containing molybdenum and cobalt, at rates of 450 and 45 mg kg^-1^ of seeds, respectively. The solution had been previously fortified with 2.0 mL kg^-1^ of the commercial liquid product “*Semia 5079*” (it was applied at 1.2 × 10^5^ cells/seed), which contain strains of *Bradyrhizobium japonicum*. Afterward, the seeds were moistened with the sugared solution at 10%, with the addition of 6.0 mL of solution to the total volume of treated seeds (1 kg), with the objective of improving the adherence of the inoculant on the seeds. The seeds were treated early in the morning and then were sown at a depth of 2.0 cm in the pots. The soil moisture was corrected during the experiment to keep it at 60% of maximum water retention capacity. Besides, two plants per pot were kept during the time course of the experiment.

Seed Ni concentration was 0.53 ± 0.02 mg kg^-1^. Fertilization was according to Malavolta et al. (1962) and Raij et al. (1997), omitting N. The following nutrients were applied (mg kg^-1^ of soil): P 200; K 150; Ca 75; Mg 15; S 50; B 0.5; Cu 1.5; Fe 5; 0.1 Mo; Zn 5. Ni was applied by means of a 2.7 mmol L^-1^ NiSO_4_⋅6H_2_O stock solution.

During the beginning of bloom – at the R1 phenological stage – (Fehr and Caviness, 1977), plants from four pots for each treatment were sampled: the third trifoliate leaf from the main stem apex (Malavolta et al., 1997), shoot, root, and root nodules were subjected to nutritional analysis. Furthermore, during the beginning of bloom the rhizosphere soil was collected. Soil that adhered to the secondary root after carefully hand agitation was considered as rhizosphere soil (Barillot et al., 2013) to determine soil-available Ni. The remaining three pots of each treatment were maintained until maturity, when seeds were collected.

### Metabolite and Enzyme Analyses

Soybean leaves – the third trifoliate leaf from the main stem apex -collected for nutritional diagnosis (Malavolta et al., 1997) were used to assessment of NR and urease activities, as well as for concentrations of organic acids: oxalic, malonic, succinic, malic, tartaric, fumaric, oxaloacetic, citric and lactic. These organic acids were also determined in soybean nodules.

To determine NR activity (Mulder et al., 1959) fresh leaf samples (0.2 g) were incubated 2 h in NaH_2_PO_4_ 50 mmol L^-1^ + KNO_3_ 200 mmol L^-1^/pH 7.4 (30°C temperature). After incubation, 1 ml from solution described above was removed and NO_2_^-^ was determined by adding 1 ml 58 mmol L^-1^ sulfanilic acid in 20% HCl to stop the reaction, followed by the addition of 1 ml 42 mm L^-1^ alpha-naphthylamine to develop a pink product by reacting with the NO_2_^-^-diazotized sulfanilate. Absorbance was determined at 560 nm (Mulder et al., 1959) and compared to a nitrite standard curve.

Urease activity was determined according to Hogan et al. (1983). Leaf samples were incubated in phosphate buffer pH 7.4 (50 mmol L^-1^) with concentration urea and n-propanol. Ammonia released was determined by adding aliquots to a mixture of two solutions, I and II (I – Phenol 0.1 mol L^-1^, sodium nitroprusside 170 μm L^-1^; II – NaOH 0.125 mol L^-1^, Na_2_HPO_4_ 12H_2_O 0.15 mol L^-1^, NaOCl_2_ – 3%). Urease activity was normalized to leaf fresh weight (μmol NH_4_^+^ g^-1^ h^-1^).

For determination of organic acids, 0.2 g of fresh plant material (leaves and root nodules), cooled to 5°C, was macerated in 20 ml 80% ethanol (V/V), the extract pH was adjusted to 2.1 (with 0.5 M H_2_SO_4_), passed through a 45 μm nitrocellulose filter and re-cooled. Immediately prior to analysis, 0.1 mL was evaporated in an N_2_ gas stream and rehydrated to the original volume. Analysis of organic acids was performed simultaneously by liquid chromatography (HPLC) over an HPLC Varian system equipped with a binary pump (ProStar 210) and a UV/Vis detector (ProStar 325) monitoring at 210 nm. An Aminex HPX-87H column (300 mm × 7.8 mm; Bio-Rad) was used. The operating conditions consisted of 0.008 N H_2_SO_4_ in the mobile phase at a flow rate of 0.6 ml min^-1^ at room temperature (25°C) and 6.687 × 10^6^ Pa pressure. Samples were filtered through a 0.45 μm pore membrane before injection; 25 μL of each samples were injected and running time was 20 min, after 10 min stabilization time. The mixed calibration standards were composed of organic acids with at least five concentrations of each. The minimum limit of detection was 2 ppm for: lactic, malonic, malic, tartaric and citric acid; 1 ppm: oxalic and succinic acid; 0.5 ppm: oxaloacetic acid; and 0.1 ppm for fumaric acid. Identification was made by comparison of peaks as a function of retention time, and concentration by peak area.

### Nutrient Analyses

Soybean leaves collected for nutritional diagnosis, grains, shoot, nodules, and root were analyzed for N and Ni concentration in of soybean. N accumulation in shoot and grains was calculated by multiplying the dry-mass by the element concentration of the respective plant part.

N concentration was determined according to the Kjeldahl method. Organic N was oxidized with sulfuric acid in the presence of catalysts and increasing temperature. Ni concentration was determined after digestion with nitric + perchloric acid (Miller, 1998), and analyzing the extract *via* ICP-OES spectrometry.

### Ni-Available on Rhizosphere Soil

Ni-available on rhizospheres soil were determined by DTPA extractant according to Lindsay and Norwell (1978).

### Microscope Analysis

Fresh root nodules were cut in the middle, examined and photographed using stereoscopic microscope Leica EZ4D^®^ camera and LAD EZ^®^ version 1.8.0. Nodule colors were analyzed by using Minolta CR-200 b colorimeter previously calibrated with white surface. Three color parameters were analyzed: L (lightness), a (interval between the red and green), b (interval between yellow and blue). Samples were measured in triplicate.

### Statistical Analysis

The results were submitted to statistical analysis using the software SAS System for Windows 6.11 (SAS 1996). The analysis of variance (ANOVA) was carried out, and based on the level of significance in the *F* test (*p* < 0.05). The data were analyzed using factorial ANOVA, followed by Tukey’s HSD test to identify significant differences among the treatments. The pearson correlation was established between root nodule colors and N-accumulation in leave diagnosis and grains.

## Results

### Ni available in Rhizosphere

Rhizosphere soil Ni available by DTPA in treatment under BCS50 was 93% higher than BCS70 (**Table 1**). This difference was clearly observed in Ni dosage of 10 mg dm^-3^ in which, the BCS50 showed Ni available 114% higher than BCS70 in the same dosage. The treatments 0.1; 0.5; 1.0 mg dm^-3^ Ni showed, respectively, 0.25; 3,75 and 6,25 times Ni available in the rhizosphere soil if compared to control treatment.

**Table 1:** Nickel concentration in the grains (at phonological stage R7) and nickel concentration in the shoot, leaves, roots and nodules, and nickel available by DTPA in rhizosphere soil in soybean plants subjected to two cation-base saturation ratio and nickel rates, assayed at phonological stage R1.

	Nickel rates (mg^.^dm^-3^)									
	0.0		0.1		0.5		1.0		10.0	
**Nickel available in rhizosphere soil (mg kg^-1^)**				
BCS50^a^	0.07	±0.00^aB^	0.11	±0.01^aB^	0.31	±0.05^aB^	0.60	±0.11^aB^	6.26	±0,70^aA^
BCS70^b^	0.09	±0.01^aB^	0.10	±0.01^aB^	0.30	±0.05^aB^	0.39	±0.03^aB^	2.93	±0,93^bA^
**Ni concentration in grains (mg kg^-1^)**				
BCS50^b^	0.34	±0.21^aC^	1.12	±0.32^aC^	5.07	±0.70^aC^	11.50	±1.65^bB^	32.17	±4.08^bA^
BCS70^a^	0.70	±0.17^aD^	1.99	±0.10^aD^	7.46	±0.41^aC^	29.88	±5.22^aB^	38.04	±1.16^aA^
**Ni concentration in leaves (mg kg^-1^)**				
BCS50^a^	0.60	±0.02^aC^	0.59	±0.07^aC^	4.67	±0.74^aB^	5.00	±0.44^aB^	19.83	±2.33^aA^
BCS70^b^	0.31	±0.23^aB^	0.62	±0.13^aB^	0.75	±0.16^bB^	0.92	±0.07^bB^	3.67	±1.35^bA^
**Ni concentration in shoot (mg kg^-1^)**				
BCS50^a^	0.47	±0.14^aC^	0.73	±0.12^aC^	1.61	±0.54^aBC^	2.44	±0.10^aB^	10.43	±1.47^aA^
BCS70^b^	0.29	±0.06^aC^	0.41	±0.04^aC^	0.79	±0.17^aC^	2.58	±1.03^aB^	6.98	±1.09^bA^
**Ni concentration in roots (mg kg^-1^)**				
BCS50^a^	0.65	±0.14^aB^	1.34	±0.21^aB^	1.46	±0.40^aB^	2.47	±0.34^aB^	20.44	±8.19^aA^
BCS70^b^	0.69	±0.17^aB^	1.34	±0.21^aB^	1.48	±0.29^aB^	1.80	±0.40^aB^	9.56	±1.28^bA^
**Ni concentration in root nodules (mg kg^-1^)**				
BCS50^a^	1.53	±0.79^aB^	2.39	±0.51^aB^	2.38	±1.05^aB^	3.37	±0.61^aB^	40.60	±7.41^aA^
BCS70^b^	0.84	±0.20^aB^	0.54	±0.29^aB^	1.65	±0.24^aB^	3.91	±1.53^aB^	28.71	±6.97^aA^

*Means followed by the same letter, lower case in columns and upper case in rows, do not differ by Tukey test at 5% of probability. ns: not significant.*

### N Concentration in Plants and N Accumulation in the Shoot and Grains

Leaf N concentration varied only with respect to Ni dosage; alteration of BCS ratio (BCS50 *versus* BCS70) had no effect (**Table 2**). At BCS50, addition of 0.1 mg dm^-3^ Ni increased leaf N concentration by 42% compared to control. It was observed that at BCS70 and 0.5 mg dm^-3^ Ni, soybean plants exhibited a 50% increase in leaf N compared to the control treatment (BCS70 and no Ni supplementation).

**Table 2:** Yield (evaluated at phonological stage R7), nitrogen concentration in the grains (at phonological stage R7) and nitrogen concentration in the shoot and leaves, nitrate reductase activity and urease in soybean plants subjected to two cation-base saturation ration and nickel rates, assayed at phonological stage R1.

	Nickel rates (mg^.^dm^-3^)									
	0.0		0.1		0.5		1.0		10.0	
**Yield (g/pot)**				
BCS50^ns^	10.79	±2.00	10.25	±2.20	11.79	±1.81	9.93	±1.70	11.53	±0.59
BCS70^ns^	10.51	±1.57	10.29	±0.74	10.32	±0.88	11.40	±0.70	11.67	±1.25
**N concentration in grains (g kg^-1^)**				
BCS50^b^	27.70	±0.93^aA^	28.18	±0.23^aA^	27.72	±0.62^bA^	28.84	±1.12^aA^	27.73	±1.20^aA^
BCS70^a^	28.23	±0.81^aB^	28.31	±0.62^aB^	31.78	±0.14^aA^	26.86	±0.73^bB^	28.50	±1.19^aB^
**N concentration in shoot (g kg^-1^)**				
BCS50^ns^	13.23	±2.51	11.34	±1.36	12.58	±1.66	13.79	±1.37	11.88	±1,63
BCS70^ns^	12.51	±2.10	13.58	±0.93	12.43	±1.25	12.88	±1.33	14.42	±1,56
**N concentration in diagnosis leaves (g kg^-1^)**				
BCS50^ns^	9.31	±2.06^B^	13.25	±1.35^A^	11.24	±0.98^AB^	10.73	±1.62^AB^	11.08	±2.07^AB^
BCS70^ns^	9.36	±1.69^B^	11.59	±1.78^AB^	14.18	±0.62^A^	9.80	±2.88^B^	10.91	±2.29^AB^
**N concentration in nodules (g kg^-1^)**				
BCS50^ns^	24.64	±2.19	19.35	±5.24	25.69	±1.18	24.41	±0.75	21.37	±1.78
BCS70^ns^	26.81	±1.59	23.82	±1.70	25.53	±2.50	27.18	±1.44	25.20	±0.45
**Leaf urease activity (μmol N-NH_4_^+^ g^-1^ h^-1^)**				
BCS50^a^	348.47	±96.53^aB^	444.30	±24.35^aA^	448.6	±36.67^aA^	421.70	±20.88^aAB^	454.30	±20.59^aA^
BCS70^b^	270.09	±46.68^bB^	331.10	±48.44^bB^	451.6	±32.47^aA^	450.30	±7.61^aA^	461.30	±23.74^aA^
**Leaf nitrate reductase activity (μmol NO_2_^-^ g^-1^ h^-1^)**				
BCS50 ^ns^	1.27	±0.38	1.13	±0.37	1.26	±0.52	0.90	±0.31	0.91	±0.46
BCS70 ^ns^	1.03	±0.32	1.30	±0.48	1.69	±0.61	1.23	±0.37	1.11	±0.35

*Means followed by the same letter, lower case in columns and upper case in rows, do not differ by Tukey test at 5% of probability. ns: not significant.*

**FIGURE 1:**
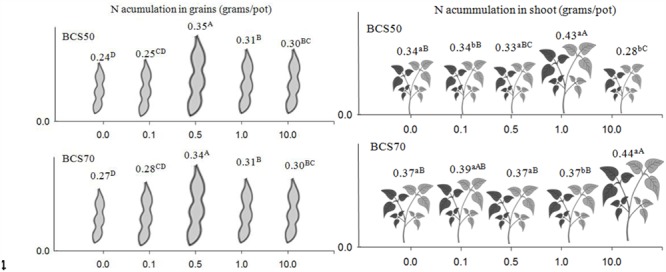
**Nitrogen accumulation in the grains (at phonological stage R7) and shoots (at phonological stage R1) in soybean plants subjected to two cation-base saturation ration and nickel rates.** Means followed by the same letter, lower case in BCS and upper case in Ni rates, do not differ by Tukey test at 5% of probability. BCS: base-cation saturation.

For grains, plants grown on BCS70, soybean seeds had greater N concentration than those from plants grown on BCS50. As in the leaves, a higher concentration of seed N was observed in plants cultured in BCS70 supplemented with 0.5 mg dm^-3^ Ni. This same treatment provided plant with more N accumulation in grains (**Figure 1**).

Furthermore, at BCS50, addition of 1.0 mg dm^-3^ Ni increased shoot N accumulation by 27% compared to control and, at BCS70, addition of 10.0 mg dm^-3^ Ni increased shoot N accumulation by 19% compared to control.

### Ni Concentration in Plants

Soybean leaf Ni concentration was greater for plants grown on BCS50 compared to plants cultivated on BCS70 for the same levels of soil Ni: At 0.5, 1.0 and 10.0 mg Ni dm^-3^ soil, leaves of plants grown on BCS50 compared to plants on BCS70 had, respectively, 6.2; 5.4 and 5.4 times greater leaf Ni concentration (**Table 1**). Ni concentration in nodules was 57% higher than in the roots, which in turn was 44% higher than the leaves and 82% higher than the shoot. Additionally, plants grown in BCS50 showed Ni-concentration 29% more in nodules, 43% in roots, 80% in leaves, and 30% in shoot, when compared to plants grown in BCS 70. Detailed results for boron (B), cupper (Cu), iron (Fe), manganese (Mn) and zinc (Zn) concentration in leaves of soybean can be found in the Supplementary Table 1. Cu, Fe and Mn were reduced by the higher BCS and, in general, Ni rates did not influence the concentration of micronutrients in soybean leaves (except for Cu in BCS50, Fe and Zn in both BCS; Supplementary Table 1).

The Ni concentration in the grains had an inverse relationship to what was observed in the leaves: Plants grown on BCS70 accumulated 36% more Ni in the grain compared to plants grown on BCS50. Grain Ni concentration increased with soil Ni rate, and the treatments did not affect the seed yield of each plant.

### Activities of NR and Urease

Interactions were not observed between the Ni levels and BCS with respect to leaf NR activity taken in soybean at phonological stage R1 (**Table 2**). Urease activity was higher in soybean leaves grown in soil with BCS50 compared to BCS70. Ni applications resulted in 20–30% increased leaf urease activity. Soybean plants grown on BCS70 showed lower activity of urease at zero (Control) and 0.1 mg dm^-3^ Ni. However, urease activity in soybean leaves from plants on BCS70 and 10.0 mg dm^-3^ Ni was 70% higher compared to the no-Ni control treatment. Urease activity taken on soybean leaves from plants on BCS50 and BCS70 and Ni rates of 0.5, 1.0, and 10 mg dm^-3^ Ni were not statistically different.

### Organic Acids

Concentrations of organic acids varied greatly depending on the tissue analyzed, to the extent that were used two different units for data presentation. Organic acids evaluated in the leaves were expressed as g kg^-1^, and for roots and nodules, as mg kg^-1^.

**FIGURE 2:**
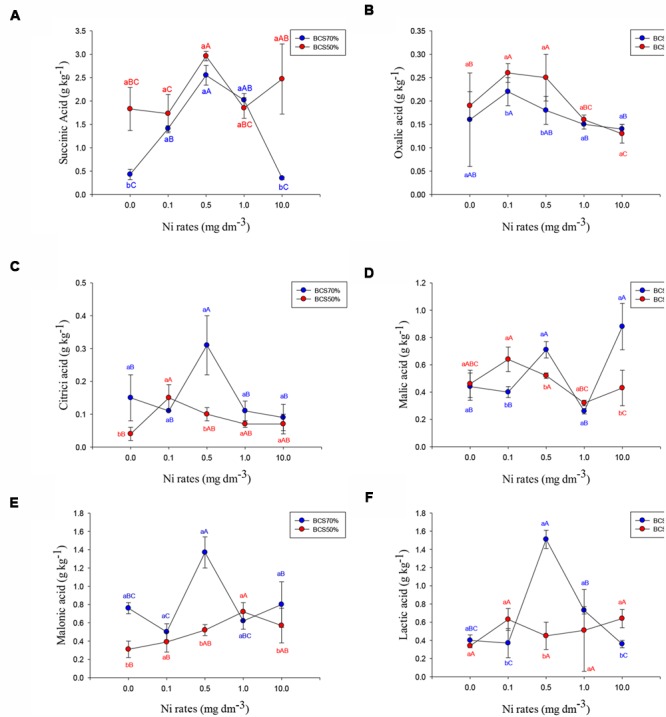
**Concentration of succinic acid **(A)**, oxalic acid **(B)**, Citric acid **(C)**, malic acid **(D)**, malonic acid **(E)** and lactic acid **(F)** in soybean leaves subjected to two base-cation saturations and nickel rates assayed at phonological stage R1.** Means followed by the same letter, lower case in BCS and upper case in Ni rates, do not differ by Tukey test at 5% of probability. BCS: base-cation saturation.

Despite of the nine organic acids evaluated in leaves (**Figure 2**), only six (succinic acid, oxalic acid, citric acid, malic acid, malonic acid and lactic acid) were influenced by the interaction of two factors (BCS ratio and Ni rate). The concentration of fumaric acid was below the minimum limit of detection for soybean. The same was true for tartaric and oxaloacetic acids in soybean.

Citric, lactic and malonic acid concentration in soybean leaves showed similar statistical results. They were not affected by Ni additions to BCS50, and they showed higher concentrations at a dose of 0.5 mg dm^-3^ Ni on the BCS70 regime. In soybean leaves, with the exception of oxalic and succinic acids, higher concentrations of organic acids were observed on BCS70. None of the organic acids evaluated showed higher concentration in the control (“0 Ni”) treatment; rather, were either lower or showed no difference.

**FIGURE 3:**
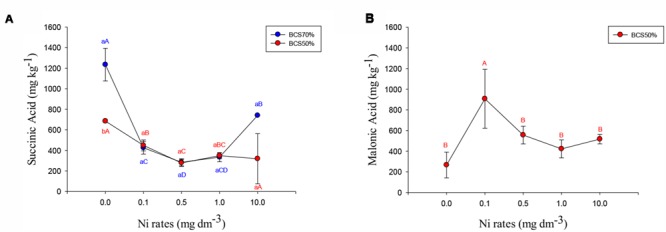
**Concentration of succinic acid **(A)** and malonic acid **(B)** in soybean nodules subjected to two cation-base saturations and nickel rates assayed at phonological stage R1.** Means followed by the same letter, lower case in BCS and upper case in Ni rates, do not differ by Tukey test at 5% of probability. BCS: base-cation saturation.

Succinic acid was the only organic acid above the detection limit across all treatments for soybean nodules (**Figure 3A**). Malonic acid was detected in soybean nodules only in the BCS70 regime (**Figure 3B**). The other evaluated acids were either below the limit of detection or were detected only in some treatments, so it is not possible to apply statistical tests. In the soybean nodules oxaloacetic acid was detected on BCS50 at doses of 1.0 mg dm^-3^ Ni (26 ± 13 mg kg^-1^); and 10.0 mg dm^-3^ (41 ± 16 mg kg^-1^); lactic acid was detected in nodules of no-Ni control plants grown under BCS70 (280 ± 106 mg kg^-1^).

### Microscope Analysis

**FIGURE 4:**
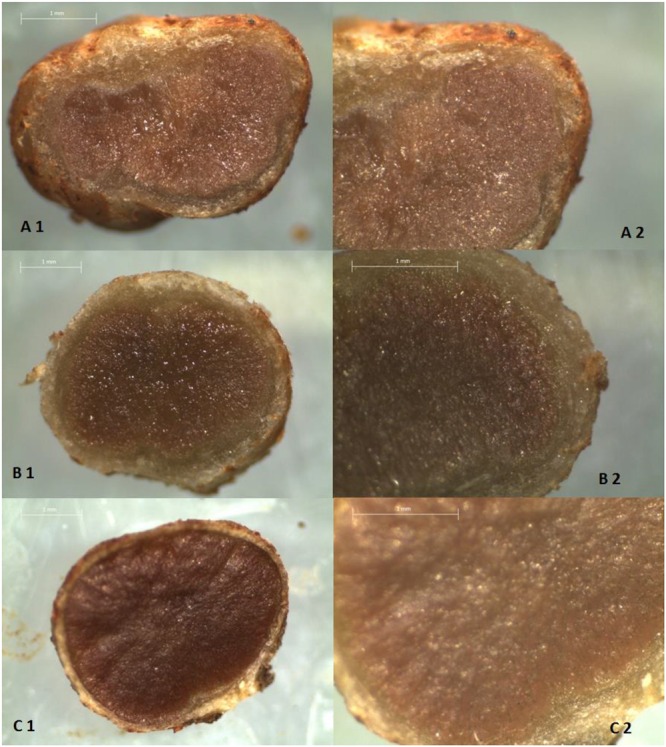
**A-1 Longitudinal section A-2 Approximate cutting of soybean nodules grown in pots with samples Alfisol with BCS50 and 0.0 g dm^-3^ of Ni.** B-1 Longitudinal section. B-2 Approximate cutting of soybean nodules grown in pots with samples Alfisol with BCS50 and 0.5 g dm^-3^ of Ni. C-1 longitudinal section. C-2 Approximate cutting of soybean nodules grown in pots with samples Alfisol BCS50 and 10.0 g dm^-3^ of Ni. Bars: 1 mm.

**FIGURE 5:**
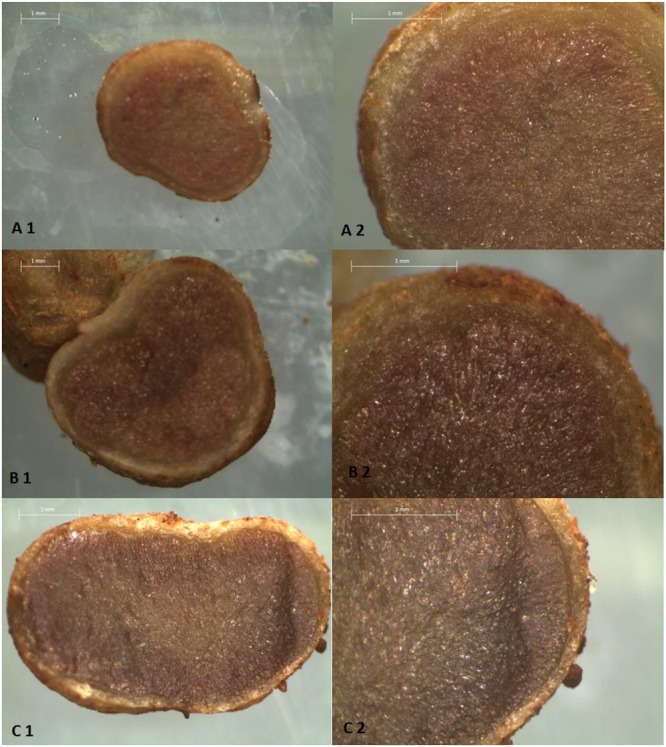
**A-1 Longitudinal section A-2 Approximate cutting of soybean nodules grown in pots with samples Alfisol with BCS70 and 0.0 g dm^-3^ of Ni.** B-1 Longitudinal section. B-2 Approximate cutting of soybean nodules grown in pots with samples Alfisol with BCS70 and 0.5 g dm^-3^ of Ni. C-1 longitudinal section. C-2 Approximate cutting of soybean nodules grown in pots with samples Alfisol BCS70 and 10.0 g dm^-3^ of Ni. Bars: 1 mm.

The color root nodules showed visual difference according with each treatment (**Figures 4** and **5**). The pearson correlation showed meaningfulness from B color nodules and N accumulation in shoots. For lightness (L) the nodules showed an inverse correlation to N accumulation in shoots and grains (**Table 3**).

**Table 3:** Correlation between color nodules and N accumulation in the shoot and grains soybean.

	Colorimeter		
	L	A	B
Shoot	-0,34^∗^	-0,11^ns^	0,39^∗^
Grain	-0,33^∗^	-0,23^ns^	-0,11^ns^

*^∗^significant at 5% probability. ns: not significant*

## Discussion

The concentration of micronutrients in soybean leaves had values considered adequate for soybeans – except for Cu that was below the optimal value, and B that was above the ideal value – according to Malavolta et al. (1997) (Supplementary Table 1). Ni concentration in soybean leaves, shoot, root, nodules and grains, was strongly influenced by soil basic cation saturation (BCS). The lower Ni concentration in soybean leaves observed in plants grown with lower Ni availability on BCS70, may halt the urease activity, N concentration, as well as the organic acids (succinic, oxalic, citric, malic, malonic) concentration. On the other hand, higher grain Ni concentration was observed in plants grown on BCS70. It was observed that the Ni concentration in grains depends not only of Ni concentration in plant (nodule, root, shoot, leaf), but also of base cation saturation that plants grew, in which it can favor Ni retranslocation, *via* phloem (Page and Feller, 2015; Deng et al., 2016). Control BCS50 plants (0.0 mg dm^-3^ Ni) exhibited seed Ni concentrations less than those of the seeds used in planting, indicating the need to apply Ni in the soil for greater concentration Ni in the grain. Additionally, Ni supply as well as Ni reserves has been pointed out for the best N nutritional status of soybean (Kutman et al., 2014). The improved performance of the plants grown in the BCS70 and the need for Ni application in soybean is evidenced by the higher concentration/accumulation of N in grains of soybean grown on BCS70 and 0.5 mg dm^-3^ Ni. Malavolta et al. (2006) had reported that healthy plants tend to accumulate more Ni in reproductive structures and Ghasemi et al. (2014) reported enhancing flowering in *Alyssum inflatum* by Ni increase. In Brazil, BCS70 was recommended by Coutinho et al. (2008) for soybean. However, the available Ni concentration of soil when employing BCS70, and no added Ni, does not appear to meet the physiological needs of these plants. These results highlight the need to establish critical levels of Ni for tropical soils according to the BCS regime. Caires et al. (2003) had reported liming limiting the availability of soil Zn, to the extent of causing Zn deficiency in soybean, while Novais et al. (1989) found Mn deficiency in soybean caused by soil liming. It is noteworthy that for a long time the available Mn concentrations in these soils were reported to be toxic. However, Fageria and Stone (2008) stated that intensive farming without restitution of micronutrients can impoverish the soil and limit yield of agricultural crops. The same can happen for Ni, notably in plants fixing atmospheric N_2_, such as soybeans (Lavres et al., 2016).

Despite there is a positive correlation between N concentration in grains and total protein content (Neves et al., 1987), the protein concentration is most often negatively correlated with grain yield, whereas the relationship between oil concentration and grain yield is positive (Rodrigues et al., 2014). In this sense, Ni supplement can be beneficial to increase the protein content, since the nickel supplementation increased the N concentration in grains.

The highest N accumulation in the leaves of soybean plants that received Ni may have occurred due to the higher efficiency of the urease activity. This enzyme is engaged in the N recycling in the leaves in subsequent senescent catabolism of arginine, producing urea in the cytosol (Witte, 2011). After the stage V6 the first leaves of the lower third of the plant begins the process of senescence (Ritchie et al., 1994), so that at the beginning of the reproductive period, there are redistribution and reallocation intense of nutrients, since the middle and upper third leaves should supply sinks like flowers and then fruits, of which they have connections (Taiz and Zeiger, 2010).

BCS70 was recommended for soybean due to its dependence on N received via symbiosis. Since almost all of the N supplied to soybean comes from biological fixation (BNF) in tropical soil conditions (Hungria and Vargas, 2000; Hungria et al., 2005, 2006; Campo et al., 2009), and since BNF is apparently carried out by seed-inoculated *B. japonicum*, there needs to be an environmental optimum for these bacteria assuming that these bacteria compete against endogenous bacteria, and represent the sole, or predominant, bacteroid population. Thus, for pH values ≤5, there is better adaptation of fungi and over a pH range of 6 to 8 there is greater development of bacteria and actinomycetes. Moreira and Siqueira (2006) pointed out that at initial pH above 5.5, as reached by BCS70, nodulation and nitrogen fixation (BNF) are favored. In this sense, the higher pH induced by the BCS70 provided a better environment for soil *Bradyrhizobium* (Barberi et al., 2004) and consequently made for more efficient symbiosis (according to the N concentration in leaves/grains). Lately, the Ni supplement in the seed was reported to advantage BNF for soybean in tropical conditions (Lavres et al., 2016). Although N concentration in root nodules were not statistically different, was observed difference in color between the nodules (**Figures 4** and **5**). This color showed positive correlation with N concentration in leaves, N accumulation either in grains or in the plant aerial parts (**Table 3**). Additionally, the intensity of pink color in the central tissue of nodules is due to leghemoglobin content, which in turn is a reliable indicator of N-fixating efficacy (Giraud et al., 2013).

The low NR activity observed in this study can be attributed to the contribution of atmospheric N_2_ on BNF, which is the main N-source for legumes. As such, fixed N is incorporated in root nodules into ureides (e.g., allantoin and allantoic acid). Thus, the formation of nitrate is probably limited. Weissman (1964), comparing N sources (ammonium and nitrate) available to soybean and sunflower, did not detect NR activity in leaves and roots of both plants when ammonium was sole N source. Furthermore, it has been stated that NR activity in leaves tend to decrease during soybean bloom period (at R1 flowering stage), whereas the activity of nitrogenase in the nodules tend to rise (Nelson-Schreiber and Schweitzer, 1986; Neves et al., 1987).

The low urease activity observed in leaves of soybean plants grown on BCS70 and low Ni dose (0 – control and 0.1 Ni mg dm^-3^) occurred as a function of pH. Since pH is crucial to Ni availability (Soares et al., 2011), increasing Ni rate on soil at BCS70, would overcome pH effects, leading to increased Ni uptake (**Table 1**) and urease activity (**Table 1**). The increase in the urease activity due to Ni supply is widely known (Gerendás and Sattelmacher, 1997; Gerendás et al., 1999; Lavres et al., 2016) as well as the effects of Ni availability as function of the soil pH (Soares et al., 2011).

The higher concentration of organic acids observed in the leaves compared to the roots nodules in this study agrees with Yang et al. (1997) who studied the effects of Ni on the concentration of organic acids in roots and leaves of grasses. The organic acid concentrations on leaves of soybean was in general higher in BCS70 with intermediate doses of Ni (e.g., 0.5 mg dm^-3^ Ni; **Figure 2**), being this condition as the ideal for plant development. At this Ni rate, the highest urease activity (**Table 2**) as well as leaves and grain N- accumulations were observed (**Figure 1**).

The increase in lactic acid in plants grown on BCS70 and 0.5 mg dm^-3^ Ni, here considered as an ideal condition for the tested crop plants, is especially puzzling. Walker et al. (1985) observed increased lactic acid in cowpea plants (*Vignia unguiculata*) deficient in Ni and attributed this effect to a metabolic response to low urease enzyme activity. Lactic acid was reported by Bai et al. (2006) as a product of glycolysis under anaerobic conditions and which may potentially occur as an indirect response of Ni deficiency. This hypothesis is not supported by these results since treatments which resulted in higher levels of lactic acid were those that provided the best traits (urease activity, concentration of N and organic acids).

The hypothesis for increased lactic acid accumulation in the treatment (BCS70 and 0.5 mg dm^-3^ Ni) considered “ideal,” is that it is result of a plant strategy for rapid energy production from a “fast metabolic pathway,” i.e., by anaerobic glucose breakdown. This hypothesis is supported by the growth stage (flowering) at which plant tissues were sampled. Because these plants have a determinate growth habit, there is great demand for energy in a short time for production of reproductive structures (flowers and fruits). Evidence for an influence of Ni on flowering period was reported by Malavolta et al. (2006) who found that half of the Ni that accumulated in the leaves, branches and flowers of orange plants was in the flowers.

The organic acids are found in the roots or rhizosphere in large concentrations as an adaptive response to a stressful environment (Yang et al., 1997; López-Bucio et al., 2000). In general, the organic acids detected in the soybean nodules were little and in low concentrations. It was also observed that the concentration of these organic acids was lower at the intermediate Ni doses (0.1; 0.5; and 1.0 mg dm^-3^ Ni), an effect contrary to that observed in soybean leaves.

## Conclusion

Soybean showed better physiological performance when grown in BCS70. The choice of BCS is crucial to establishing optimal Ni supplementation for crop plants. Redistribution of Ni from the leaves to the grains is variable not only in function of Ni doses applied, but also the BCS level.

Soybean needs Ni supplementation for better urease activity, BNF, increase organic acids, N accumulation (therefore, increase protein concentration) and Ni concentration. This was evident in the increase of urease activity according to Ni rates; the positive correlation between the colors of the nodes and the N accumulation of the leaves; increased succinic acid, cictric, lactic and malonic in diagnostic leaves; the increase in Ni concentration in soybeans.

In addition, the Ni concentration of the soybeans derived from the control treatment was less than the seed used in the experiment; so that may occur decrease on Ni concentration in the grains without Ni supplementation. Consequently, according to the results obtained in this study, N metabolism in the fixating N-plants can be impaired or improved by Ni availability.

## Author Contributions

All authors listed (FGM, JDB, EFS, FF, WTLS, JCP, and JL), have made substantial, direct, and intellectual contribution to the work, and approved it for publication.

## Conflict of Interest Statement

The authors declare that the research was conducted in the absence of any commercial or financial relationships that could be construed as a potential conflict of interest.
