# Supplementary material for: Nickel Availability in Soil as Influenced by Liming and Its Role in Soybean Nitrogen Metabolism
Source: Front Plant Sci. 2016 Sep 8;7:1358. doi: 10.3389/fpls.2016.01358 (PMC5014873; doi:10.3389/fpls.2016.01358)
Supplement: Supplementary file 1 [file Table_1.DOCX]

Supplementary data

Supplementary Table 1. Concentration of micronutrients in soybean leaves subjected to two base-cation saturations and nickel rates assayed at phonological stage R1

|  |  | Ni rates | | | | | | | | | | | | | | |
| --- | --- | --- | --- | --- | --- | --- | --- | --- | --- | --- | --- | --- | --- | --- | --- | --- |
|  |  | 0.0 mg dm^-3^ | | | 0.1 mg dm^-3^ | | | 0.5 mg dm^-3^ | | | 1.0 mg dm^-3^ | | | 10.0 mg dm^-3^ | | |
|  |  | ---------------------------------------------mg kg^-1^ --------------------------------------------- | | | | | | | | | | | | | | |
| Cu | BCS50^b^ | 2.83 | ± 1.67^A^ | 2.94 | | ± 1.00^A^ | 4.62 | | ± 1.69^A^ | 3.65 | | ± 1.53^A^ | 5.36^A^ | | ± 0.54^A^ |  |
|  | BCS70^a^ | 4.95 | ± 0.71^AB^ | 2.70 | | ± 2.01^B^ | 5.84 | | ± 0.38^A^ | 5.25 | | ± 0.43^A^ | 5.78^A^ | | ± 0.76^A^ |  |
|  |  |  |  |  | |  |  | |  |  | |  |  | |  |  |
| Fe | BCS50^a^ | 210.56 | ± 24.11^C^ | 400.25 | | ± 17.86^A^ | 290.36 | | ± 27.96^B^ | 247.49 | | ± 17.19^BC^ | 260.17 | | ± 63.45^BC^ |  |
|  | BCS70^b^ | 216.82 | ± 48.87^A^ | 273.09 | | ± 67.83^A^ | 277.22 | | ± 76.84^A^ | 218.74 | | ± 61.45^A^ | 212.77 | | ± 46.51^A^ |  |
|  |  |  |  |  | |  |  | |  |  | |  |  | |  |  |
| Mn | BCS50^a^ | 95.44 | ± 9.27 | 101.51 | | ± 19,05 | 112.47 | | ± 34.76 | 109.72 | | ± 20.78 | 92.93 | | ± 8.37 |  |
|  | BCS70^b^ | 77.86 | ± 6.12 | 77.97 | | ± 15,19 | 85.51 | | ± 29.48 | 100.72 | | ± 39.66 | 59.46 | | ± 2.91 |  |
|  |  |  |  |  | |  |  | |  |  | |  |  | |  |  |
| Zn | BCS50^ns^ | 30.63 | ± 1.62^B^ | 46.42 | | ± 6.05^B^ | 67.70 | | ± 9.67^A^ | 44.80 | | ± 12.10^B^ | 38.78 | | ± 10.55^A^ |  |
|  | BCS70^ns^ | 36.51 | ± 2.77^B^ | 42.51 | | ± 3.54^AB^ | 53.19 | | ± 7.92^A^ | 44.84 | | ± 7.87^AB^ | 42.71 | | ± 4.43^AB^ |  |
|  |  |  |  |  | |  |  | |  |  | |  |  | |  |  |
| B | BCS50^ns^ | 54.05 | ± 11.31 | 63.28 | | ± 12.24 | 65.35 | | ± 5.54 | 72.00 | | ± 15.51 | 55.63 | | ± 16.04 |  |
|  | BCS70^ns^ | 66.28 | ± 13.71 | 60.45 | | ± 8.36 | 71.19 | | ± 5.82 | 64.58 | | ± 6.72 | 64.05 | | ± 6.00 |  |
|  |  |  |  |  | |  |  | |  |  | |  |  | |  |  |
| Ni | BCS50^a^ | 0.60 | ± 0.02^aC^ | 0.59 | | ± 0.07^aC^ | 4.67 | | ± 0.74^aB^ | 5.00 | | ± 0.44^aB^ | 19.83 | | ± 2.33^aA^ |  |
|  | BCS70^b^ | 0.31 | ± 0.23^aB^ | 0.62 | | ± 0.13^aB^ | 0.75 | | ± 0.16^bB^ | 0.92 | | ± 0.07^bB^ | 3.67 | | ± 1.35^bA^ |  |

(F1: BCS%) (F2: Ni rates) (Interaction F1 x F2). F = Factor. Cu(*)(**)(^ns^); Fe (*)(**)(^ns^); Mn (**)(^ns^)(^ns^); Zn (^ns^)(**)(^ns^); B(^ns^)(^ns^)(^ns^); Ni (**)(**)(**). ** significant at 1% probability; * significant at 5% probability. ns: not significant. Lowercase: column. Capital: line.
